# Progress in Nanostructured Mechano-Bactericidal Polymeric Surfaces for Biomedical Applications

**DOI:** 10.3390/nano13202799

**Published:** 2023-10-20

**Authors:** S. P. S. N. Buddhika Sampath Kumara, S. W. M. Amal Ishantha Senevirathne, Asha Mathew, Laura Bray, Mohammad Mirkhalaf, Prasad K. D. V. Yarlagadda

**Affiliations:** 1 School of Mechanical, Medical and Process Engineering, Faculty of Engineering, Queensland University of Technology (QUT), Brisbane, QLD 4000, Australia; buddhika.naidelage@hdr.qut.edu.au (S.P.S.N.B.S.K.); s2.senevirathne@qut.edu.au (S.W.M.A.I.S.); asha.mathew@unisq.edu.au (A.M.); laura.bray@qut.edu.au (L.B.); 2 Australian Research Council Training Centre for Multiscale 3D Imaging, Modelling, and Manufacturing, Queensland University of Technology (QUT), Brisbane, QLD 4000, Australia; 3 Centre for Biomedical Technologies, Queensland University of Technology (QUT), Brisbane, QLD 4000, Australia; 4 School of Engineering, University of Southern Queensland, Springfield, QLD 4300, Australia; 5 Centre for Materials Science, Queensland University of Technology (QUT), Brisbane, QLD 4000, Australia

**Keywords:** antibacterial surfaces, nanofabrication, antimicrobial, polymeric implants, nanostructured surfaces

## Abstract

Bacterial infections and antibiotic resistance remain significant contributors to morbidity and mortality worldwide. Despite recent advances in biomedical research, a substantial number of medical devices and implants continue to be plagued by bacterial colonisation, resulting in severe consequences, including fatalities. The development of nanostructured surfaces with mechano-bactericidal properties has emerged as a promising solution to this problem. These surfaces employ a mechanical rupturing mechanism to lyse bacterial cells, effectively halting subsequent biofilm formation on various materials and, ultimately, thwarting bacterial infections. This review delves into the prevailing research progress within the realm of nanostructured mechano-bactericidal polymeric surfaces. It also investigates the diverse fabrication methods for developing nanostructured polymeric surfaces with mechano-bactericidal properties. We then discuss the significant challenges associated with each approach and identify research gaps that warrant exploration in future studies, emphasizing the potential for polymeric implants to leverage their distinct physical, chemical, and mechanical properties over traditional materials like metals.

## 1. Introduction

Bacterial cells usually attach to surfaces, colonise, and form a matrix of polysaccharide material called biofilms. Bacteria can adhere to many different surfaces, including human tissues, metals, and polymeric surfaces [1]. The bacteria that form biofilms are highly resistant to disinfectants, antiseptics, and other antimicrobial agents that are normally effective in their planktonic state [2,3,4]. Moreover, overuse of antibiotics has led to an era where bacteria are becoming increasingly resistant to antibacterial agents, making it more difficult to treat infections [4,5]. The discovery of the mechano-bactericidal response to nano-topography found on insect wings, such as cicadas and dragonflies, has inspired the development of nanostructured topography on synthetic materials, which can reduce or inhibit the growth of bacteria on their surfaces. Most of the studies on artificial antibacterial micro/nanostructured surfaces were based on materials like ceramics, metals, and alloys, because of their superior mechanical and thermal properties that lead to potential applications in medical and industrial sectors. Metals are among the most used engineering materials and are employed extensively as biomaterials [6,7]. However, they can cause inflammation, bone loss, and imaging artifacts in computed tomography (CT) scans and magnetic resonance imaging (MRI) due to their magnetic properties, low flexibility, and low biocompatibility compared to biocompatible polymers [8,9]. High cost and density characteristics also make them less effective in specific medical implants such as cardiovascular implants, absorbable implants, paediatric implants, and cochlear implants [9,10,11,12,13,14,15]. Biocompatible polymers are attractive alternatives that can be used in such applications due to ease of processing, low weight, high biocompatibility, and biodegradability [16,17,18,19]. The methods for developing nanostructured bactericidal surfaces have been reviewed in the recent literature [20,21,22,23,24]. However, a comprehensive and critical overview of the techniques developed for polymers is yet missing. This review provides such an overview showing the progress in the field, identifying research gaps, and discussing opportunities and possible future directions.

## 2. Polymers Used in Biomedical Applications

Many biocompatible polymers are used in medical implants with some demonstrating high biocompatibility [25]. The most commonly used biocompatible polymers are polyglycolic acid (PGA) [26,27], poly(lactic-co-glycolic acid) (PLGA) [26,28,29,30], polycaprolactone (PCL) [26,31,32], polyurethane (PU) [33], polyvinyl alcohol (PVA) [34,35,36], silicone [33,37,38], polylactic acid (PLA) [26,39,40,41,42,43,44], polypropylene (PP) [45,46], and polymethyl methacrylate (PMMA) [47,48,49]. Each polymer has distinct advantages and limitations in various biomedical applications, as mentioned in Table 1. For instance, PMMA is a frequently used polymer for dental implants due to its non-degradability and high strength while PLA is used in bone tissue engineering applications due to its biodegradability and osteoblast stimulation [39,50]. Moreover, polyetheretherketone (PEEK) and polyetherketoneketone (PEKK) are emerging biomedical materials with high biocompatibility, thermal stability, and non-degradability with excellent mechanical properties which are suitable for various biomedical applications [51,52]. 

**Figure 1:**
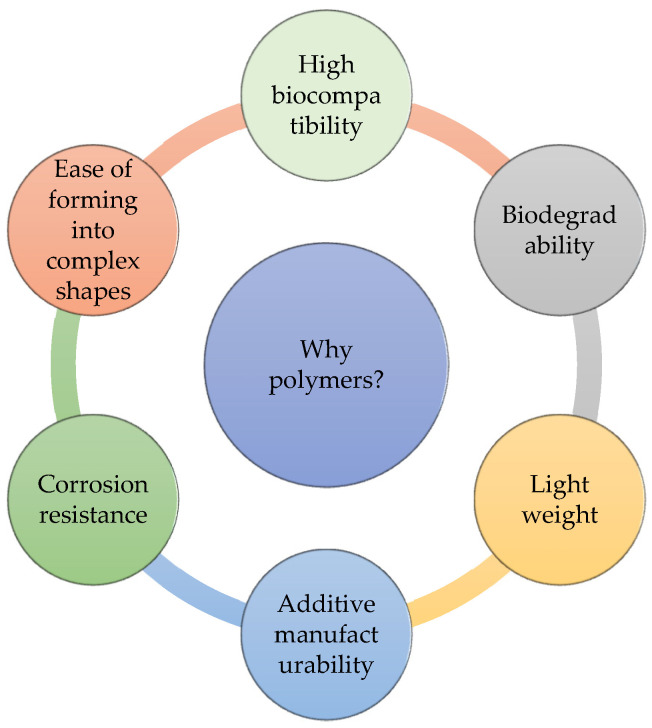
Driving factors to choose a polymer as implant material. Compared to other materials polymers provide some favourable characteristics for biomedical implant applications, which is essential in the biomedical field for successful implant in in vivo applications.

In such applications, one of the most concerning issues is implant failures due to bacterial infections as described in the Introduction section. Bacteria colonise various implant surfaces, such as dental implants, catheters, and orthopaedic implants [53]. Furthermore, antibiotic resistance directly contributes to implant failures due to the formation of biofilms on these surfaces [54,55]. In response to this concern, numerous studies have been undertaken to develop physical and chemical methods aimed at reducing bacterial colonisation [56,57]. To implement these strategies successfully, the choice of implant material plays a pivotal role in developing surfaces that are bactericidal and/or antifouling. Among various materials, polymers offer advantageous characteristics for biomedical implant applications. These attributes are highly considered in the biomedical field, particularly for ensuring a successful implant surgery in in vivo conditions (Figure 1).

**Table 1:** Polymers used in biomedical applications. Table abbreviations: Polydimethylsiloxane—PDMS; Polyethylene terephthalate—PET; Polytetrafluoroethylene—PTFE.

Polymer	Characteristics, Physical, and Mechanical Properties	Biomedical Applications	Advantages	Limitations	Ref.
PGA	Biodegradable, biocompatible, tensile stress: 890 MPa, density: 1.5 g/cm^3^, melting point: 225–230 °C, glass transition temperature: 35–40 °C	Tissue engineering applications in bone, tendon, cartilage, tooth, and spinal regeneration; nerve grafts; absorbable sutures	Stimulates cartilage regeneration; 3D-printability; high tensile strength	High friction coefficient and “binds and snags” when wet, high brittleness, high degradation causes inflammatory response	[26,27,58,59,60]
PLGA	Biodegradable, bioadsorbable, biocompatible, tensile stress: 3.4 MPa, density: 1.2 g/cm^3^, melting point: dependent on the percent composition (PLA, PGA), glass transition temperature: 40–60 °C	Therapeutic tools; drug delivery; tissue engineering	Stimulates osteoblasts; 3D-printability	Release of acidic byproducts leads to inflammation, degrade due to hydrolysis, poor strength	[26,28,29,30,61]
PCL	Biodegradable, bioadsorbable, biocompatible, tensile stress: 12.8 MPa, density: 1.15 g/cm^3^, melting point: 60 °C, glass transition temperature: −60 °C	Dental splints; drug delivery; tissue engineering	Stimulates osteoblasts; 3D printable; slow degradation rate; low cost in 3D printing due to low melting point; high biocompatibility	Poor mechanical properties; low cell adhesion	[26,31,62]
PU	Can be biodegradable or non-biodegradable based on chemical composition, non-bioabsorbable, biocompatible, tensile strength: 34.5–56 MPa, density: 1.23 g/cm^3^, melting point: 163 °C, glass transition temperature: −35 °C	Drug delivery; catheters, pacemaker leads insulation, vascular prostheses, heart valves, cardiac assist devices (cardiovascular applications)	High durability; high toughness; good biostability; low cost	Environmental stress cracking; material degradation in vivo; metal ion oxidation	[16,63,64,65]
PP	Non-biodegradable, non-bioabsorbable, biocompatible, tensile stress: 28 MPa, density: 0.9 g/cm^3^, melting point: 170 °C, glass transition temperature: −25 °C	Sutures; scaffolds (ligament or tendon repair); meshes for hernia and pelvic organ repair; heart valve structure, oxygenator and plasmapheresis membranes, finger joint prosthesis	High melting point; less toxic; low cost	Limited biocompatibility; poor strength	[16,33,66,67,68]
PVA	Biodegradable, biocompatible, tensile stress: 40–90 MPa, density: 1.26 g/cm^3^, melting point: 228 °C, glass transition temperature: 85 °C	Wound dressings, drug delivery, targeted-tissue transportation systems; soft biomaterial implants.	High chemical and thermal stability; non-toxic	Weak hydrogel endurance in high temperature; relatively weak polymer; limited biocompatibility; degrades due to hydrolysis	[34,69,70,71,72]
Silicone or PDMS	Non-absorbable, non-biodegradable, biocompatible, hydrophobic, tensile stress: 2–10 MPa, density: 0.97 g/cm^3^, melting point: 228 °C glass transition temperature: ~120–123 °C	Oxygenator membrane; tubing; shunts; prostheses; heart peacemaker leads; heart valve structures; burn dressing	Chemically inert; low toxicity; thermal stability; high biocompatibility	Prone to damage; non-durable; contamination of monomers; low mechanical strength	[16,47,63]
PLA	Biodegradable, bioabsorbable, biocompatible, tensile stress: 21–60 MPa, density: 1.21–1.25 g/cm^3^, melting point: 150–160 °C, glass transition temperature: 60–65 °C	Bone tissue engineering; drug delivery; plates, screws, pins, and wires in bone fixation; bio-absorbable implants; sutures in dermatology; drug-eluting stents	High biocompatibility; stimulates osteoblasts; less brittle; one of the highly used 3D-printable materials; degradation products are also non-toxic to humans and the environment.	Low mechanical strength	[26,33,39,40,66,67,73]
PMMA	Non-degradable, biocompatible, tensile stress: 48–76 MPa, density: 1.2 g/cm^3^, melting point: 130–180 °C, glass transition temperature: 80 °C	Dental implants; bone cement; lenses; drug delivery	One of the hardest thermoplastics with high scratch resistance; high mechanical strength	Less biocompatibility; high curing temperature; does not support osteointegration; causes necrosis effect	[16,74,75]
PEEK	Non-degradable, biocompatible, tensile stress: 84 MPa, density: 1.4 g/cm^3^, melting point: 343 °C, glass transition temperature: 143 °C	Dental implants; knee implants; spine implants; cranioplasticity; hip replacement; anterior plate fixation; heart valves; face reconstructions	High biocompatibility; 3D-printable; light weight; compatible with hydroxyapatite (natural bone tissue materials) hence substitute to metallic implants; stable at high temperatures; mechanical stability	Low thermoformability; bioinert (does not promote tissue integration); complex and costly manufacturing process	[76,77,78]
PEKK	Non-degradable, biocompatible, tensile stress: 115 MPa, density: 1.3 g/cm^3^, melting point: 363–386 °C, glass transition temperature: 162 °C	Dental implants; crown and bridge in dentistry; endodontic post; removable denture framework; restorative and prosthetic applications	High biocompatibility; 3D-printable; light weight; high mechanical strength; excellent chemical resistance	Bioinert (does not promote tissue integration); more complex and costly manufacturing process than PEEK	[52,79]
PET	Non-degradable, high biocompatibility, tensile stress: 75–100 MPa, density:1.38 g/cm^3^, melting point: 255–265 °C, glass transition temperature: 85 °C	Sutures; heart valves; surgical meshes; scaffolds; urinary and bloodstream catheters; commercial vascular prosthesis	3D-printable; cost effective; excellent chemical resistance	Bioinert (does not promote tissue integration)	[80,81,82]
PTFE	Non-degradable, biocompatible, tensile stress: 30.5 MPa, density: 2.175 g/cm^3^, melting point: 327 °C, glass transition temperature: 127 °C	Vascular graft prostheses; heart patches; stapes prosthesis	High mechanical strength; chemically inert	Difficult to 3D-print	[16,83]
Chitosan	Biodegradable, biocompatible, tensile stress: 32.2 MPa, density: 0.20–0.38 g/cm^3^, melting point:105 °C, glass transition temperature: 75 °C	Antitumor drug delivery; protein and peptide drug delivery; gene delivery; antibiotic delivery; polyphenol delivery; wound healing applications	Antimicrobial; anti-inflammatory; antifungal; nontoxicity; antitumor activity; antioxidant activity	Low mechanical strength; significant variations of properties based on the source of material	[84,85,86,87,88,89,90]

## 3. Mechanism of Bacteria Adhesion on Surfaces

Bacteria are present in different environments, such as animals, soil, plants, fresh water, and air [91]. Bacterial adhesion refers to the capability of bacteria to attach to a range of surfaces, such as human tissues, medical implants, polymers, metals, and glasses [92]. This process is vital for bacterial colonisation and is achieved through a complex process that involves few stages with multiple factors. As an overview, it is affected by the distinct characteristics of bacteria such as motility, cell wall structure and appendages (flagella, pili, and curli), exposure duration to the surface, amount of nutrients, coaggregation, cohesion, and bacterial density [92,93,94]. Bacteria colonisation occurs in two stages: (1) prior attachment stage, known as the primary stage or reversible adhesion, and (2) post-attachment stage, known as the secondary stage or irreversible adhesion [95]. During the reversible (volatile) adhesion, once the bacteria reach a certain proximity from the surface, their adhesion depends on the superposition of attractive and/or repulsive forces such as electrostatic, van der Waals, hydrophobic, and hydrodynamic forces [95], whereas the attraction of the bacteria to the surface is high during this stage and occurs within few minutes [96]. The majority of bacteria carry a predominantly negative surface charge [97,98], especially in the initial growth stage [98], and tend to selectively bond with surfaces that are positively charged [99]. However, if the environment (where the bacteria and surface are located) is in high ionic condition, then this electrostatic interaction will be reduced due to the charge screening effect (neutralizing) caused by oppositely charged ions in the environment [93,99]. In the second stage, adhesion becomes irreversible (permanent) without any need for physical or chemical intervention, firmly anchoring the organism to the surface within several hours [95,99]. Similar to the primary stage, van der Waal interactions are involved, which are between the outer cell wall and the surface. Moreover, polysaccharides and proteins play a crucial role in the transition from reversible to irreversible cell attachment, whereas the irreversible attachment is mostly dominated by the production of extracellular polymeric substances (EPSs) [92,99,100]. EPSs consist of polysaccharides, proteins, extracellular DNA, and lipids. EPSs, which are released by cells in biofilms adhered to surfaces, protect against mechanical damage and shear generated by flow [99]. Notably, within biofilms, EPSs exhibit a non-homogeneous distribution pattern among cells [101]. In the biofilm, an EPS offers various advantages to the cells. These benefits include adhesion, protection, and structural support. Specifically, the aggregative polysaccharides function as molecular glue, facilitating the adhesion of bacterial cells both to each other and to surfaces [102]. In addition to these factors, the surface characteristics of the substrate, including factors like surface charge density, wettability, roughness, stiffness, and surface architecture, are also regarded as significant factors that influence the initial adhesion of bacteria to surfaces [94]. Once these bacterial cells colonise a surface, they can create numerous problems such as infections. Many different methods are used to mitigate bacterial colonisation.

## 4. Antimicrobial Strategies

### 4.1. Chemical Bactericidal Strategies

Bactericidal chemicals play a major role in mitigating bacterial colonisation. Upon attachment, the planktonic bacterial cells begin clustering and start to form biofilms. Biofilms are highly resistant to antiseptics, antibiotics, and immune killing [103,104,105,106]. These biofilms create adverse effects on medical implants such as infections and continual inflammatory reactions, that can lead to implant failure [103,107,108,109]. Many researchers investigated chemical antibacterial strategies to avoid this issue. A common strategy is the widespread use of bactericidal and bacteria-repelling chemical agents, such as antibiotics and antiseptics [110]. Under the same category, antimicrobial surface coatings are more popular in many antibacterial applications [111,112,113,114,115,116,117,118]. Metal (Ag, Au, Cu, Zn) nanoparticles [113,119,120], metal oxide (ZnO, MgO, CuO, TiO_2_) nanoparticles [120,121], graphene family materials [122,123,124,125], fullerene [126,127], and carbon nanotubes [125] are frequently used as coatings [128] and additives for composites [129] in implants. For instance, Rezic et al. developed an antibacterial coating on PLA polymer using different types of commercially available nanoparticles such as Ag, Al_2_O_3_, Au, Pt, TiO_2_, Y_2_O_3_, ZnO, and ZrO_2_ [56]. This coating was bactericidal against Gram-positive *Staphylococcus aureus* (*S. aureus*) strains. Moreover, Patil and colleagues developed a silver-nanoparticle-embedded polystyrene polymer composite via thermal annealing and soft moulding process [130]. This composite showed an excellent bactericidal effect on both Gram-negative *Escherichia coli (E. coli)* and Gram-positive *S. aureus* bacteria.

The interaction of these nanoparticles with bacterial cells leads to several effects, including the inhibition of enzymes, deactivation of proteins, induction of oxidative stress, disruption of electrolyte balance, and alterations in gene expression levels resulting in lysing the bacteria [131]. These materials demonstrate excellent bactericidal efficacy. But the underlying antimicrobial mechanisms might induce a cytotoxicity effect for human cells [132,133,134,135]. For instance, Berardis et al. and Guan et al. studied toxicity assessments of ZnO nanoparticles on human colon carcinoma cells and human hepatocyte cell lines via toxicity assay methods. They found that these nanoparticles cause a reduction in human cell viability, presence of inflammatory biomarkers, DNA, and mitochondrial damage [136,137]. Moreover, Haase et al. studied the toxicity of silver nanoparticles using human leukemia cells and concluded that the silver nanoparticles cause elevated lactate dehydrogenase and a reduction in cell viability [138]. Moreover, the biggest threat to chemical-based antibacterial agents is the ability of bacteria to evolve into antimicrobial-resistant strains [139,140]. Given the widespread usage of antibacterial chemicals, they have the potential to be redundant due to antimicrobial resistance, less susceptibility of bacteria in biofilms, and potential cytotoxicity. These challenges necessitate alternative methods that are not prone to those threats.

### 4.2. Physical/Mechanical Bactericidal Strategies

#### 4.2.1. Natural Bactericidal Nanostructured Surfaces

**Figure 2:**
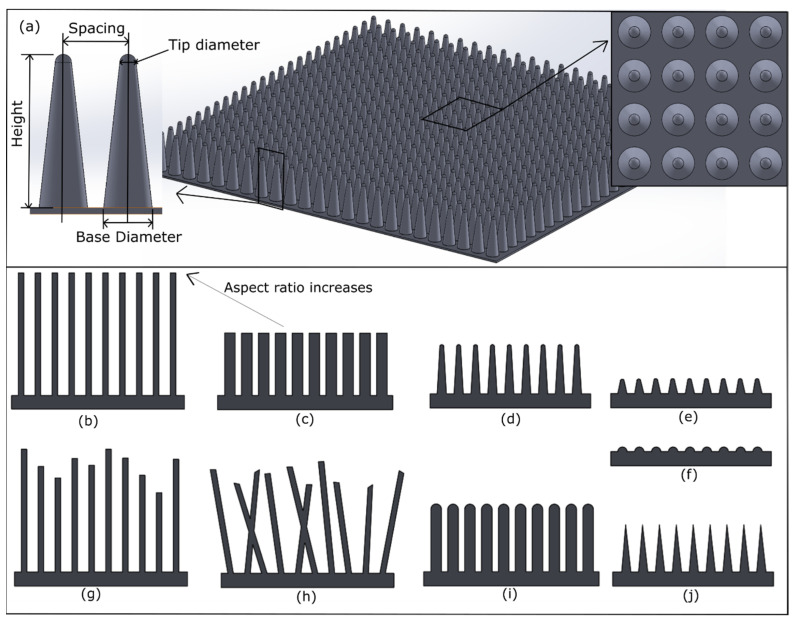
(**a**) Major nanofeature parameters; (**b**–**j**) different shapes of nanofeatures, type of nanoarchitectures (uniform (**b**–**f**,**i**,**j**) and random (**g**,**h**)), and aspect ratio.

During the last decade, patterns with impressive bactericidal properties have been found on shark skin, gecko skin, lotus leaves, and the wings of cicadas, damselflies, and dragonflies [23,141,142,143]. These wings contain nanostructured surfaces that can prevent the growth of biofilms and kill a variety of bacteria by generating high bactericidal levels to different bacteria strains, as depicted in Table 2 [141,144]. For example, Ivanova et al. showed that cicada wing surfaces can eliminate Gram-negative *Pseudomonas aeruginosa* (*P. aeruginosa*) bacteria within 3 min of contact [145]. Interestingly, highly patterned or regularly arrayed nanoscale pillars on cicada wings are effective against Gram-negative bacteria, while the random nanofeatures or sigmoidal nanoarchitecture of dragonfly wings can kill both Gram-negative and Gram-positive cells [141,146,147,148,149]. Tripathy et al. reviewed that cicada wings are proficient at effectively lysing Gram-negative bacteria but not Gram-positive bacteria. The reason is due to the Gram-positive bacteria’s thick peptidoglycan cell wall, which is approximately 4 to 5 times thicker than that of Gram-negative bacteria [24]. The various nanopatterns comprise either patterned or random arrays of surface features (Figure 2b,h), and are easily distinguished by the height, tip diameter, base width, and spacing of their individual surface features [57,150], as shown in Table 2 and Figure 2.

**Figure 3:**
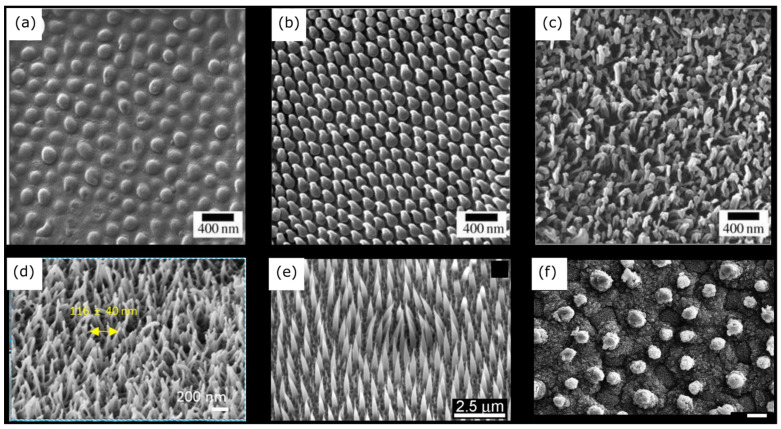
Micrographs of natural bactericidal nanostructured surface topographies. (**a**) Helium ion micrograph of a periodical cicada wing (*Magicicada septendecim*) showing hemispherical features. (**b**) Helium ion micrograph of an annual cicada wing (*Tibicen tibicen*) showing spherically capped conical features. (**c**) Helium ion micrographs of a common sanddragon dragonfly wing (*Progomphus obscurus*) showing spherically capped cylindrical features with a high aspect ratio. Adapted with permission from ref. [154]. (**d**) Scanning electron microscopy (SEM) micrograph (tilted 45°) showing the self-organised free-standing and clustered nanopillar arrays on a black damselfly wing (*C. haemorrhoidalis*). Adapted with permission from ref. [155]. (**e**) SEM micrograph of natural gecko skin (*Strophurus williamsi*). Adapted from ref. [110]. (**f**) SEM micrograph of the surface of a natural lotus leaf (scale: 10 μm). Adapted with permission from ref. [156]. Copyright© 2015, American Chemical Society. Dragonfly (**c**) and damselfly (**d**) wings comprise similar non-uniform (random) nanoarchitectures with different heights and orientations. However, the nanofeatures in gecko skin (**e**) comprise a high aspect ratio and high spacing nanofeatures compared to all the other natural nanostructures.

**Figure 4:**
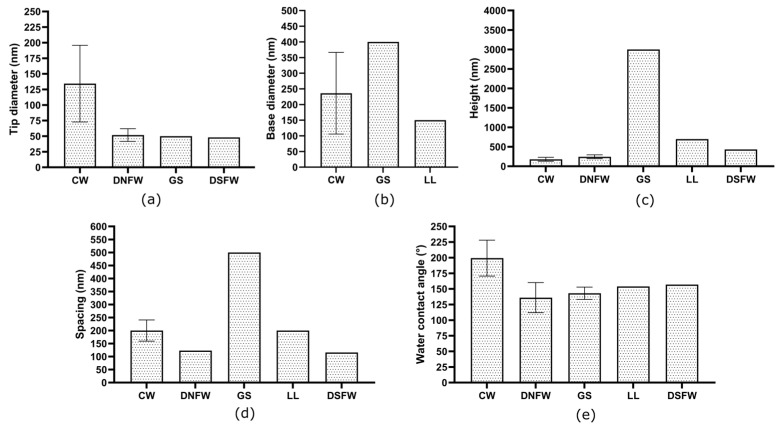
Deviation of nanofeature parameters such as (**a**) tip diameter, (**b**) base diameter, (**c**) height, (**d**) spacing, and (**e**) surface wettability among the different natural bactericidal nano-topographies listed in Table 2. The mean values of each parameter are plotted with standard deviation error bars. Graph abbreviations: cicada wing—CW; dragonfly wing—DNFW; gecko skin—GS; damselfly wing—DSFW; lotus leaf—LL.

The cicada wing species demonstrate ordered nanoarchitecture while dragonfly, damselfly, and gecko skin present random or hierarchical nanoarchitecture (Table 2 and Figure 3). All these surfaces are composed of nanopillars while gecko skin and lotus leaves are composed of spinules and nanotubes, respectively. The spinules are wire-like features with high aspect ratio having a height around 3000 nm [110]. There is a significant difference between the height of nanofeatures in gecko skin and other natural bactericidal nano-topographies (Figure 4). Moreover, cicada wing nano-topographies are bactericidal to Gram-negative bacteria species such as *P. aeruginosa* and *P. fluorescens,* while dragonfly wings, gecko skin, and damselfly wings are bactericidal to both Gram-negative (*P. aeruginosa*, *E. coli*, and *P. gingivalis*) and Gram-positive (*S. aureus*) bacteria. This bacterial cell rupture is caused by the prolonged suspension of the bacterial cell membrane on the nanostructured surface, which causes the membrane to stretch beyond its elastic limit [24,150,151,152,153]. It is also noticeable that the tip diameter is low when it comes to dragonfly wing, gecko skin, and damselfly wing architecture compared with cicada wings. Hence, it results in creating high stress on the bacterial cells to lyse the bacteria. As per the literature results included in Table 2, it is shown that there is an effect on bactericidal activity from the nanostructure pattern type for the different bacteria species. The absence of threats of antimicrobial resistance development and antibacterial tolerance due to biofilm formation makes bactericidal nano-topographies more appealing for both medical and industrial applications.

**Table 2:** Natural antibacterial nanostructured surfaces.

Scheme	Nanofeature Type	Nanofeature Arrangement	Dimensions of the Nanofeature				Wettability	Antimicrobial Activity	Ref.
			Tip Diameter (nm)	Base Diameter (nm)	Height (nm)	Spacing (nm)			
Periodical cicada wing (*Magicicada* ssp.)	Hemispherical shape nanofeatures	Ordered	167	167	83.5	252	~80°	Lethal to *S. cerevisiae* fungus	[154]
Dog day annual cicada (*Tibicen* ssp.)	Nanoneedles (spherically capped, conical, nanoscale pillars)	Random/hierarchical	57	104	183	175	132°	Bactericidal to *P. aeruginosa* (Gram-negative)	[154]
Cicada wing (*Psaltoda claripennis*)	Nanopillars	Ordered	60	100	200	170	~159°	Bactericidal to *P. aeruginosa* (Gram-negative)	[145]
Cicada wing (*Megapomponia intermedia*)	Tubular nanofeatures (nanopillars)	Ordered	156	~312	241	165	~136°	Bactericidal to *P. fluorescens* (Gram-negative)	[101]
Cicada wing (*Cryptotympana Aguila*)	Tubular nanofeatures (nanopillars)	Ordered	159	~318	182	187	~113°	Bactericidal to *P. fluorescens* (Gram-negative)	[101]
Cicada wing (*Ayuthia spectabile*)	Tubular nanofeatures (nanopillars)	Ordered	207	~414	182	251	~96°	Bactericidal to *P. fluorescens* (Gram-negative)	[101]
Sanddragon dragonfly wing (*Progomphus obscurus*)	Nanograss	Random/hierarchical	53	-	241	123	119°	Bactericidal to *P. aeruginosa* (Gram-negative), *S. aureus* and *B. subtilis* (Gram-positive)	[154]
Dragonfly wing (*D. bipunctata*)	Nanograss	Random/hierarchical	50–70	-	240	-	153°	Bactericidal to *P. aeruginosa* (Gram-negative), *S. aureus* and *B. subtilis* (Gram-positive)	[146]
Dragonfly wing (*Orthetrum villosovittatum*)	Nanopillar	Random/hierarchical	37 and 57	-	189 and 311	-	-	Bactericidal to *E. coli* (Gram-negative)	[148]
Gecko skin (*Strophurus williamsii*)	Spinules	Random/hierarchical	50	~400	3000	500	>136°	-	[110]
Gecko skin (*Lucasium steindachneri*)	Spinules	Random/hierarchical	-	-	2000–4000	~500	150°	Bactericidal to *Streptococcus mutans* (Gram-positive) and *Porphyromonas gingivalis* (Gram-negative)	[102,131]
Lotus leaves (*Nelumbo nucifera*)	Nanotubules	Random/hierarchical	-	100–200	300–1100	~150–250	154°	Bactericidal to *E. coli* (Gram-negative)	[157]
Damselfly wing (*Calopteryx haemorrhoidalis*)	Nanopillars	Random/hierarchical	~48	-	~433	116	157°	Bactericidal to *P. aeruginosa* (Gram-negative) and *S. aureus* (Gram-positive)	[149]

#### 4.2.2. Artificial Nanostructure Fabrication Methods in Polymers

To generate different morphologies of polymeric nanostructured surfaces, many studies were conducted using different chemical and mechanical methods. Nanoimprinting lithography (NIL) [57,132], electron beam lithography (EBL) [141,158], reactive ion etching (RIE) [159,160], colloidal lithography [161,162], laser-based lithography techniques, and anodic aluminium oxide template (AAOT) [141,163,164] are polymer nanostructured surface fabrication methods which are used in the literature (Table 3). The NIL, EBL, and AAOT processes demonstrate high nanofeature parameter controllability. However, limitations like high fabrication cost and multiple process steps make them non-facile and inefficient [141,158,163,165].

##### Reactive Ion Etching

**Figure 5:**
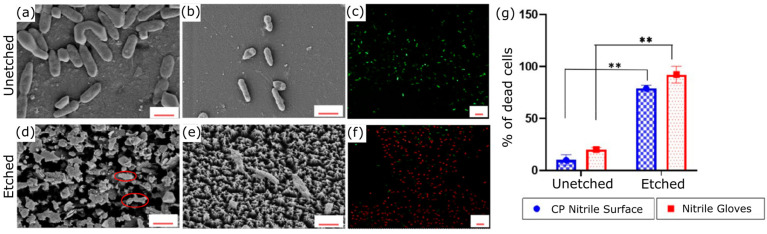
(**a**,**b**) SEM micrographs (scale: 1 µm) of *P. aeruginosa* bacteria on unetched polymer surfaces; (**d**,**e**) SEM micrographs (scale: 1 µm) of lysed *P. aeruginosa* bacteria on etched polymer surfaces using RIE; (**c**,**f**) live/dead confocal micrographs (60×) of unetched and etched polymer surfaces with *P. aeruginosa* bacteria; (**g**) % of dead *P. aeruginosa* cells on each unetched and etched polymer surface. ** indicates significance for *p* < 0.01. Adapted with permission from ref. [160]. Copyright© 2023, American Chemical Society.

Reactive ion etching (RIE) is perhaps the most frequently used method to develop non-polymer nanostructured surfaces, and produces high-aspect-ratio nanostructures by mimicking cicada and dragonfly nanostructures [159,166]. It is based on high energetic ion which is bombarded into the substrate coated with a photoresist pattern mask to remove material from the surface which is unexposed to photoresist, resulted in nanostructured surfaces [167,168]. Ivanova et al., Hasan et al., Roy et al., and Ganjian et al. have used the RIE process to fabricate high-aspect-ratio antibacterial nanostructured surfaces on non-polymer materials such as black silicon and black titanium [146,169,170]. However, limited literature related to polymer surfaces was found. Kayes et al. developed a nanostructured surface on PP polymer via maskless RIE (without using a photoresist pattern mask) method under different plasma gas conditions, and achieved a bactericidal activity on *E.coli* bacteria [171]. Moreover, Patil et al. was successful in obtaining more than 85% of bactericidal efficacy against *P. aeruginosa* bacteria on commercially pure nitrile (CPN) and nitrile gloves without using a mask in RIE [160]. Moreover, the etching time, type of gas, gas pressure, plasma gas flowrate, and plasma power are crucial parameters in this process to optimise the nanostructures to obtain the required bactericidal efficacy. Another study involving maskless RIE on the PET polymer fabricated bactericidal nanostructures against *P. aeruginosa*. Nevertheless, the same etched surface did not demonstrate bactericidal efficacy against *S. aureus* bacteria [172]. Interestingly, the generated surfaces are random arrays of nanofeatures as depicted in Figure 5d,e. This process is comparatively a facile method due to the absence of the photoresist mask. Yet, there is no evidence in the literature that maskless RIE is used to fabricate nanostructures on polymer surfaces which are effective in lysing both Gram-negative and Gram-positive strains. 

##### Colloidal Lithography

**Figure 6:**
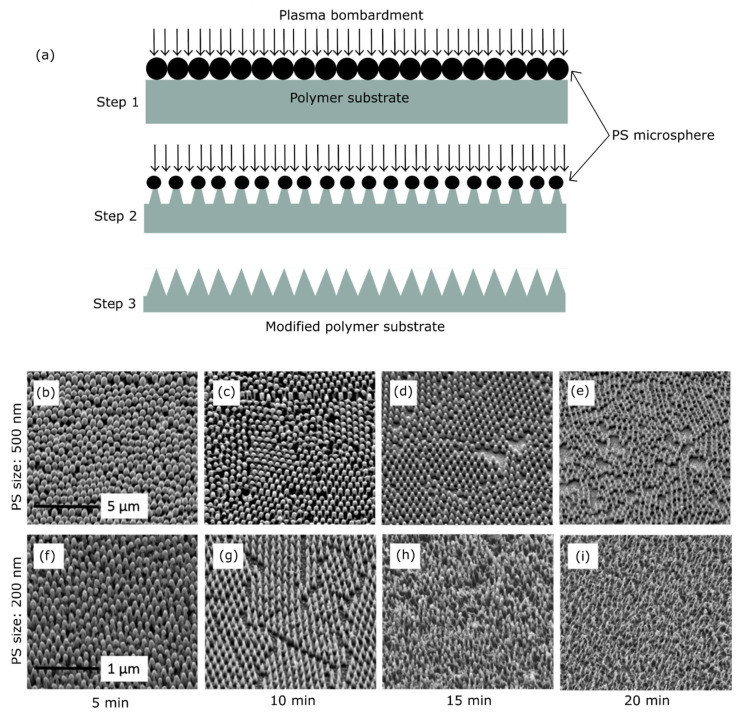
(**a**) Colloidal lithography process steps. Step 1: Spin-coated PS microspheres on the polymer substrate. Step 2: Halfway etched microspheres and polymer substrate. Step 3: Completely etched microspheres and developed nanocone structures on the substrate. (**b**–**i**). SEM micrographs of PET nanocone structures fabricated using 500 nm (**b**–**e**) and 200 nm (**f**–**i**) PS microspheres at different etching times. Images (**b**–**i**) adapted with permission from ref. [161]. Compared to the maskless RIE process, colloidal lithography is capable of producing different nanoarchitectures via mimicking cicada (**b**–**g**), dragonfly (**h**,**i**), and damselfly (**h**,**i**) wings.

Colloidal lithography is a similar process to RIE which uses nano- or microspheres instead of a photoresist mask. Distinct nanostructured surfaces are obtained by controlling the micro/nanosphere size, plasma gas pressure and flow rate, and etching time [161,162,173]. Hazel et al. fabricated bactericidal PET nanocones via a combination of colloidal lithography and RIE [161]. To fabricate the nanocones, initially the polystyrene (PS) microspheres were spin-coated as a 2D hexagonally close-packed array, as depicted in Figure 6 (step 1). Nanostructure size is dependent on the size of the microsphere, and 200 nm and 500 nm sized microspheres were used to produce uniform nanocones in this work. Oxygen plasma is bombarded on the PS coated substrate with relevant power and flow rate to etch the PS and substrate. Further etching will reduce the size of the PS microsphere and remove the substrate material while taking the PS microsphere as a mask (Figure 6 (step 2)). For each of the PS microsphere sizes, different etching times were performed to obtain distinct nanofeatures on the surface with different height, tip diameter, and spacings, as depicted in Figure 6b–i. The researchers were able to produce nanostructures with a 20 nm tip width and 400 nm height which were bactericidal to Gram-negative *E. coli* and *Klebsiella pneumoniae* (*K. pneumoniae*).

Mo et al. fabricated different types of nanofeatures (nanopillars, micropillars, microcones) on PEEK polymer using PS microspheres as the mask [162]. The fabricated nanofeatures were lethal to *E. coli* bacteria. A similar process has been used as the one depicted in Figure 6a. However, the researchers used two gases such as Ar and O_2_. Using Ar in colloidal lithography can produce nanotube-like features via anisotropic etching (etching in vertical direction) without decreasing the size of the microspheres. On the other hand, using O_2_ as the plasma gas can produce nanocone features while etching material isotropically (etching in both vertical and horizontal directions) by decreasing the size of the microspheres. Hence, this process can be effectively used to fabricate nanostructured surfaces on polymers via controlling the parameters like microsphere size, plasma power, gas flowrate, and etching time. Colloidal lithography can produce a uniform array of nanostructures with varying nanofeature sizes more effectively than the RIE procedure.

##### Nanoimprinting Lithography

**Figure 7:**
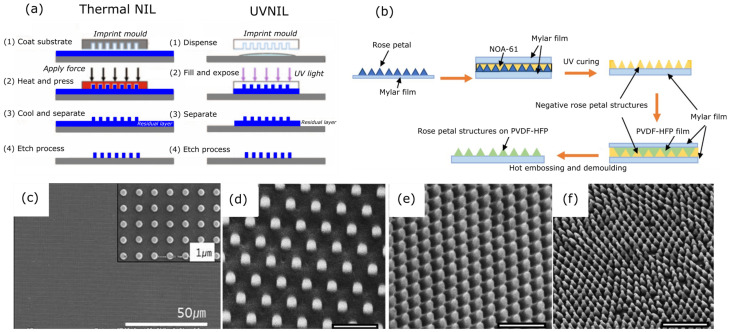
(**a**) TNIL and UVNIL process steps. Adapted with permission from ref. [175]. (**b**) Fabrication steps of rose petal mimetic structures on PVDF-HFP films. Adapted with permission from ref. [179]. Copyright© 2023, American Chemical Society. (**c**) SEM micrographs of nanopatterned thermal shrinkage films via UVNIL process. Adapted with permission from ref. [181]. (**d**–**f**) SEM micrographs (30° tilted, scale bar: 1 µm) of nanostructure made on PMMA surface with different spacings ((**a**): 600 nm, (**b**): 300 nm, (**c**): 200 nm). Adapted with permission from ref. [57].

NIL is a high-throughput fabrication approach for producing nanostructures. It entails transferring a pattern from a mould to a deposited polymer. A liquid polymer layer, known as a “resist”, is placed on the substrate surface and mechanically pressed with a silicon stamp, creating a mould pattern imprint on the polymer substrate. The mould is then removed to reveal the polymer impression, and a RIE procedure is used to remove the polymer resist (Figure 7), resulting in the final nanostructure [174,175]. The process can be divided into two types: thermal nanoimprinting lithography (TNIL) and ultraviolet nanoimprinting lithography (UVNIL) [175]. In the TNIL process, the pattern is made using both pressure and increased temperature (Figure 7a), which is often higher than the polymer’s glass transition temperature [176]. However, the mould removal process cause more damage to the nanostructures. The UVNIL process reduces the structural deformation during the demoulding process in the TNIL method. UVNIL uses UV radiation to crosslink the polymeric nanostructures (Figure 7b) [177,178]. However, some researchers have used a combination of both processes to get the maximum advantage of producing highly bactericidal nanostructured surfaces [179,180]. 

Oopath et al. created surfaces that resemble rose petals and have antibacterial properties using a combination of TNIL, UVNIL, and the hydrothermal technique [179]. Initially, they used NIL to imitate the microstructures found on rose petals on the surface of a PVDF-HFP film, as shown in Figure 7b. The hydrothermal approach was then used to grow ZnO nanostructures on the PVDF-HFP film, resembling the structure of rose petals. As shown in Table 3, the experimental findings showed that PVDF-HFP films mimicking rose petals had about 100% bactericidal efficiency against both strains of bacteria. 

Dickson et al. also used NIL to construct nanopillars inspired by cicada wing structures on a PMMA substrate (Figure 7d–f). The PMMA film was applied onto the glass substrate using a spin coating method, followed by an annealing procedure on a hot plate at 100 °C prior to the imprinting stage. Different samples were acquired via distinct approaches to obtain various sizes of nanopillars (Table 3). PDMS nano hole moulds, a nickel stamp, and natural cicada wings were used in the NIL process to imprint the pattern on PMMA surfaces [57].

Shung et al. developed a nano-in-microstructured hierarchical superhydrophobic surface using a combination of UVNIL and RIE [181]. A PDMS mould with nanostructures was fabricated using a silicon mould. Then, the UV-curable polymer resist was imprinted using the prepared PDMS mould. Later, this nanostructured polymer layer was transferred into a thermal shrinkage film with an annealing process and RIE to create the final nanostructure (Figure 7c). The created nanostructured polymer film was superhydrophobic with a water contact angle of 150°. The researchers harnessed these surfaces to create superhydrophobic surfaces, primarily for self-cleaning purposes. While the bactericidal properties of these surfaces have not been experimentally evaluated, the nanofeature characteristics seem promising for potential mechano-bactericidal effects, with respect to the mechano-bactericidal nanofeature parameter ranges in Figure 4.

Even though NIL demonstrates high controllability in nanostructure dimensions, the multiple steps in the process lead to a high cost.

##### Laser-Based Lithography Techniques

**Figure 8:**
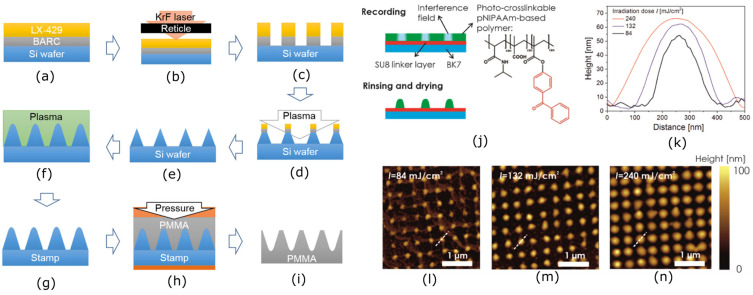
(**a**–**i**) Laser-based lithography process assisted with TNIL. Adapted from ref. [176]. (**j**) Schematic view of the crosslinking of pNIPAAm-based hydrogel polymer via arrays of interference laser beams. (**k**) Change of nanofeature parameters with irradiation dose. (**l**–**n**) AFM micrographs of pNIPAAm-based hydrogel polymer nanofeature parameters under different irradiation doses. Images (**j**–**n**) adapted with permission from ref. [190].

In laser-based lithography techniques, a laser beam or set of beams is used to create nanopatterns on different material substrates via a photosensitive material (typically a photoresist). Laser lithography, laser interference lithography, and femtosecond laser lithography are key methods used in the literature for fabricating nanostructured surfaces [176,182,183,184]. The laser lithography process uses a laser beam to directly produce a pattern on the photosensitive material. Resists used in this process can have either negative or positive tones, and the region on the resist that is exposed can be crosslinked or chain-scissioned [185]. The patterned photoresist material acts as a mask on the target substrate, and plasma etching usually follows in order to etch the unexposed area of photoresist to obtain the final nanostructure on the substrate. Kim et al. produced a nanostructured bactericidal and superhydrophobic surface on PMMA polymer using the KrF laser lithography process with dry etching, thermal oxidation, high-density plasma chemical vapour deposition, and TNIL, as depicted in Figure 8a–i [176]. BARC and LX-429 photoresists were coated on a Si wafer, and the initial nano pattern was produced using KrF laser beam. Then, a dry etching process was used with a combination of Cl_2_ and HBr gases to etch the Si wafer by taking the produced photoresist nanopattern as a mask. Subsequently, a nanocone structure was obtained, as depicted in Figure 8e. Later, the high-density plasma chemical vapour deposition process used to develop nanopillars on Si, and the TNIL process replicated the pattern on PMMA with dimensions of 250 nm (base diameter), 490 nm (height), and 300 nm (spacing) (Figure 8i). The fabricated surface exhibited bactericidal properties against *P. aeruginosa* and *E. coli* bacteria. In the early stages of attachment, which occurred during the first 1–4 h of bacterial incubation, most adhered *E. coli* cells were lysed, primarily attributable to the mechano-bactericidal effect, when compared to a flat substrate. Conversely, there was no substantial bactericidal impact on *P. aeruginosa* during this initial attachment phase. However, over a period of 2–7 days, a significant portion of both bacteria lysed due to the inhibition of adhesion and bacterial growth because of the surface’s superhydrophobic nature.

Laser interference lithography (LIL) creates detailed patterns on a substrate using the interference pattern of coherent laser beams. Complex interference patterns are formed by adjusting the angles and wavelengths of the laser beams, resulting in high-resolution patterns on the substrate [184,186,187,188]. During the process of fabricating polymer nanostructured surfaces, the initial photoresist pattern can be made using the LIL method with high-resolution patterns due to the interference of multiple beams rather than using the conventional laser lithography processes [189].

Quilis et al. fabricated arrays of thermoresponsive poly(*N*-isopropylacrylamide) (pNIPAAm)-based hydrogel nanostructures with a gold nanoparticle array for applications in highly sensitive chemical and biological sensing [190]. The four-beam UV laser interference lithography (UVLIL) method was used to pattern the photoresist on the pNIPAAm hydrogel, and the hydrogel was etched using the dry etching process to obtain the final nanostructure, as depicted in Figure 8j. This resulted in nanostructures with 132 nm (diameter), 50 nm (height), and 463 nm (spacing) dimensions (Figure 8k). Even though the spacing is too high compared to diameter and height, these methods can be used in the development of mechano-bactericidal nanostructures on polymers. Interestingly, Valle et al. fabricated microstructures on polystyrene polymer surfaces using the LIL process [191]. These microstructures took various forms, including line and pillar-like patterns as well as lamella shapes. The researchers examined bacterial attachment, using *S. aureus*, to assess the antibacterial properties in both static and fluid flow conditions. Surprisingly, the line and pillar-like microstructures seemed to promote bacterial adhesion, while the lamella-shaped patterns reduced bacterial adhesion under both static and flow conditions. Furthermore, the researchers primarily demonstrated an anti-adhesion effect as a key contributor to the antibacterial activity of these microstructures. Additionally, they proposed that these microstructures provided a mechano-bactericidal effect inspired from the microfeatures found on shark skin.

To conclude, the laser-based lithography technique is an extended version of the NIL process. Given the high precision of the nanofeatures, the various steps employed in the process make it a non-facile method in commercial implant fabrication. However, LIL can also be used as a facile method without using NIL and other supportive processes like etching as per the study of Valle et al. Moreover, to make such surfaces mechano-bactericidal, nanofeature parameters should be carefully controlled as per Figure 4.

##### Anodic Aluminium Oxide Template

**Figure 9:**
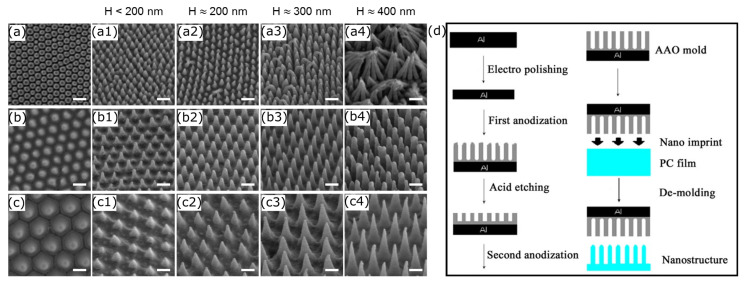
(**a**–**c**) SEM micrographs (scale: 200 nm) of fabricated nanostructures via AAOT on PC surface: (**a**) 100 nm, (**b**) 170 nm, and (**c**) 300 nm of interpillar spacing. 30° tilted SEM images of nanopillars (**a_1_**–**a_4_**,**b_1_**–**b_4_**,**c_1_**–**c_4_**) with different heights (H). Adapted with permission from ref. [195]. Copyright© 2020, American Chemical Society. (**d**) Nanostructure fabrication using AAOT. Adapted with permission from ref. [192].

To create well-ordered nanofibers, AAOT is used as a template to recreate fine nanopores employing a molten polymer. Nanostructure fabrication via AAOT consists of several steps, as depicted in Figure 9d. First anodization, acid etching, and second anodization are the key steps of producing the template. The AAOT process is used to fabricate the template which is required to use in the NIL process to impart the nanostructure to a polymer material [192]. During the initial anodization process, electoral density distribution is formed over the thin aluminium sheet. Then, the acid etching is used to remove the initial aluminium oxide layer to form an organized barrier layer to produce nano pores in an ordered manner. Finally, a secondary anodizing process follows to allow the developed pores to penetrate into the original aluminium sheet. Temperature, voltage, and the pH level of the anodising electrolyte play a crucial role of determining the pore depth, spacing, and diameter [193,194].

Cui et al. modified the polycarbonate (PC) substrate surface using AAOT-assisted wet etching and hot embossing [195]. This is similar to the procedure depicted in Figure 9d. The ability to change the geometric parameters (height, diameter, and spacing of nanopillars) of the nanostructure more efficiently (Figure 9a–c) than other manufacturing methods, such as NIL, RIE, and colloidal lithography, is an essential component of this nanostructure fabrication process. As mentioned in Table 3, the researchers fabricated bactericidal nanostructures with a bactericidal efficacy of more than 95% against *E. coli* bacteria. Moreover, the interaction between bactericidal efficacy and geometric parameters were also studied in this work, and the researchers concluded that high efficacy is obtained at 200 nm of nanopillar height with smaller cap diameters. Also, as per the study, there was an optimum value (170 nm) for the spacing which influence to have more bactericidal efficacy.

Zhang and colleagues devised a hybrid nano-topography via a mechano-bactericidal mechanism using an AAOT assisted technique [196]. They employed a layer-by-layer assembly method to create this structure, involving the sequential application of tannic acid (TA) and iron ions (Fe^3+^) through a dip-coating process. Polymeric nanopillars were crafted by forming a polymeric resin from diglycidyl ether of bisphenol A resin (DGEBA), polyether amine resin (D230), and ethyl acetate using the AAOT process. The coating of TA and Fe3+ on these nanopillars exhibited a significant photothermal effect resulting in enhanced antibacterial properties in addition to its mechano-bactericidal effect. These hybrid nanopillars demonstrated exceptional efficacy in lysing *P. aeruginosa* and *S. aureus* bacteria, with a bactericidal rate exceeding 99%. It is worth noting that the photothermal coating can have inherent cytotoxicity, but the developed surface exhibited excellent compatibility with mammalian cells (more than 88% cell viability) in in vitro studies.

Similar to NIL and laser-based lithography methods, AAOT involves a series of process steps and is primarily suitable for creating nanostructures on flat surfaces due to the difficulties involved in mould removal of NIL on curved surfaces. As AAOT utilizes the NIL process, any drawbacks associated with NIL also apply to this method.

##### Electron Beam Lithography (EBL)

Lithography is a nanofabrication method which uses light or focused particle beams to remove material from a substrate and obtain nanostructures [197]. EBL and focused ion beam (FIB) are the two main categories of the particle beam lithography (PBL) process [198]. Particles can be identified as electrons and ions (Ga+, He+, Ne+). In EBL, high-energy electrons are employed, whereas in focused ion beam (FIB) milling, high-energy ions are utilized. EBL is a maskless lithography process which uses an electron gun to fabricate nanoscale patterns on a substrate [197]. The electron beam produced by the gun either images (in SEM) a surface or fabricates a previously deposited resist on a substrate. The resist that is exposed can be crosslinked or chain-scissioned [185]. Then, exposed and nonexposed resist regions are selectively removed via a solvent. While the covered regions are protected, the exposed areas can be further processed for etching or thin-film deposition [199]. PMMA polymer is a common positive e-beam resist whose solubility changes when exposed to an e-beam [185,200]. Moreover, polyacrylic acid (PAA), polyethylene glycol (PEG), and hydrogen silsesquioxane (HSQ) are the mostly used resist materials in the EBL process [201]. 

Kallas et al. produced nanopillars on polycarbonate polymers using a combination of the UV-NIL, dry-etching, and EBL process [201]. The developed nanostructures were bactericidal against *E. coli* bacteria. The EBL process is used to make the pattern for the UVNIL process. The pattern was made from hydrogen silsesquioxane (HSQ) resist. Compared to photolithography processes, EBL provides minimum nanofeature size as fine as ~2 nm while photolithography fabricates around ~50 nm [180]. However, the direct writing of patterns by scanning electron beam is a slow process with low throughput. As a result, EBL is utilised for photomask preparation for photolithography or the direct writing of low-volume and small-area patterns.

##### Hydrothermal Synthesis

The hydrothermal synthesis process develops a high-temperature and high-pressure environment in a closed Teflon-lined stainless steel reactor [202]. During this process, the sample (or implant) is submerged completely in alkaline solution to perform a reaction to achieve nanostructures. Because of its reliability, environmental friendliness, simplicity, low cost in comparison to other procedures, and flexibility for material morphology control, hydrothermal synthesis is the most versatile nanostructured surface fabrication method for metals, ceramics, and non-flat complex geometry surfaces [159,166,203]. As per the literature relevant to hydrothermal synthesis on titanium surfaces, it can resemble the nanofeatures of cicada wings or dragonfly wings with high bactericidal efficacy [159,166,204]. Hence, theoretically this method can be used to fabricate mechano-bactericidal nanostructures on 3D implants. For instance, Jaggessar and colleagues fabricated bactericidal nanostructures on 3D titanium surfaces via the hydrothermal synthesis process, and were successful in obtaining 91% more efficiency in anti-adhesion compared to 2D surfaces against *S. aureus* bacteria, showing the same or higher bactericidal efficacy than 2D surfaces [205]. 

**Figure 10:**
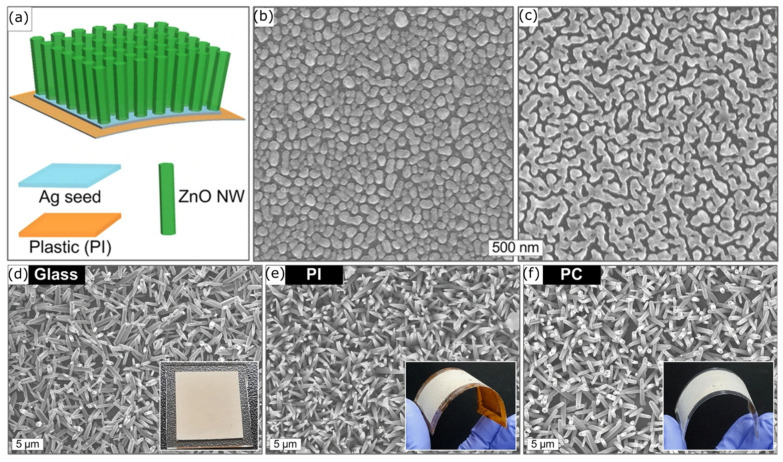
(**a**) Schematic drawing of ZnO nanowire formation on plastic substrate. (**b**,**c**) SEM micrographs of formation of nanoporous morphology of Ag ink coating on plastic substrate. (**d**–**f**) SEM micrographs of grown ZnO nanowires on glass (**d**), PI (**e**), and PC (**f**) substrates. Adapted from ref. [206].

On the other hand, due to the low melting point and low glass transition temperature of polymers, it is challenging to use the hydrothermal synthesis process to develop nanostructures on polymer surfaces. Hence, using the hydrothermal synthesis process for polymers has been overlooked in the bactericidal nanostructured surface field. However, Yoo et al. created ZnO nanowires on flexible plastics such as PET, PC, and polyimide (PI) using a low-temperature (approx. 90 °C) hydrothermal synthesis method, as depicted in Figure 10a [206]. After coating the flexible plastic substrate with Ag ink solution, the Ag layer was transformed into a nanoporous layer at 130 °C (Figure 10b,c). The hydrothermal reaction was processed inside the $Zn^{2+}$ ion solution of this Ag-coated plastic substrate. Finally, as shown in Figure 10d–f, ZnO crystallised on the Ag-seeded substrate at 90 °C after 9 h. This nano-topography is similar to the mechano-bactericidal nano-topography developed by Bhadra et al. [159] and Zhao et al. [203]. Even though there are a few studies that used the hydrothermal synthesis process to develop nanostructures via metal oxides on polymer substrates, no study has yet been reported in the literature for using hydrothermal synthesis to fabricate micro/nanostructured surfaces on pure polymers.

**Table 3:** Summary of polymer nanostructured surface fabrication methods. Table abbreviations: Poly(ethylene glycol) dimethacrylate—PEGDMA; Poly(vinylid ne fluoride-co-hexafluoropopylene)—PVDF-HFP; Height—H; Tip diameter—D_t_; Base diameter—D_b_; Diameter—D; Spacing—S; Width—W; Pillar density—PD; Pitch—P; Aspect ratio—AR; Roughness—R.

Method	Substrate Material	Supportive Processes	Feature Type	Nanofeature Parameters		Bactericidal Effect (Max. Efficiency %, Incubation Time in Hours)	Ref.
NIL	PMMA	Silicon mould preparation	Nanopillars	H	210 nm, 300 nm	Lethal to *E. coli* bacteria (~50%, 24 h)	[57]
				D_t_	70 nm, 190 nm, 215 nm		
				D_b_	100 nm, 130 nm, 380 nm		
				S	170 nm, 320 nm, 595 nm		
	OrmoStamp (glass)	AAOT, deep RIE	Nanopillars	H	200, 300, 400 nm	Lethal to *S. aureus* bacteria (~100%, 0.5 h)	[207]
				D	70, 80 nm		
				PD	40 µm^2^, 70 µm^2^		
	PEGDMA	RIE	Nanoneedle	H	300 nm	Lethal to *E. coli* bacteria (N/A)	[208]
				D_t_	50 nm		
				D_b_	200 nm		
				P	500 nm		
	PVDF-HFP	Hydrothermal method	Array of micropapillae with nanofold structures	D (papillae)	20 µm	Lethal to Gram-positive *S. agalactiae*: Micropapillae (~97%, 12 h), Nanopillars (100%, 12 h); Gram-negative *E. coli,* Micropapillae (~100%, 12 h), Nanopillars (100%, 12 h)	[179]
				D (nanofold)	300–400 nm		
				D	300 nm		
				P	600 nm		
	PC	RIE, EBL	Nanopillar	H	25 nm	Lethal to *E. coli* (N/A)	[201]
				D_b_	40 nm		
				S	100, 200, 500 nm		
Laser Lithography	PMMA	Thermal oxidation, RIE, chemical vapour deposition, TNIL	Nanopillars	H	450 nm	Lethal to *P. aeruginosa* (~70%, 8 h) and *E. coli* (~70%, 8 h)	[176]
				D_b_	250 nm		
				AR	3		
LIL	PS	None	Both pillar-like and lamella-shaped patterns	H	2 µm	Lethal and anti-adhesion under flow conditions to *S. aureus*	[191]
				W	3 µm		
				S	3 µm		
Colloidal lithography	PET	RIE	Nanocones	H	530–350 nm	Lethal to *E. coli* (~30%, 1 h) and *K. pneumoniae* (~30%, 1 h)	[161]
				D_t_	20–300 nm		
				D_b_	55–380 nm		
				AR	1–7		
	PEEK	RIE	Nanopillars, nanocones, micropillars, micro-cones	H	350 nm	Lethal to *E. coli*: Nanopillars (>95%, 12 h), Micropillars (~90%, 12 h), Micro-cones (>75%, 12 h)	[162]
				P	200, 250 nm		
				D	100, 50 nm		
AAOT	Polymer made from DGEBA and E230	Wet etching, dip coating	Nanopillars	H	500–600 nm	Lethal to *P. aeruginosa* (~99%, 3 h) and *S. aureus* (~99%, 3 h)	[196]
				D_t_	100 nm		
				D_b_	350 nm		
	PC	Hot embossing and wet etching	Nanopillars	H	143–408 nm	Lethal to *E. coli* bacteria (>95%, 3 h)	[195]
				D_t_	26.7–33.4 nm		
				D_b_	66.3–154.3 nm		
				S	100–307.8 nm		
RIE	PP	None	Nanofibrils	H	0.5, 1 µm	Lethal to *E. coli* (~99.6%, 24 h)	[171]
				D	30, 40 nm		
	CPN	None	Nanopillars	H	1–1.4 µm	Lethal to *P. aeruginosa* (~80%, 24 h)	[160]
				R	0.92–1.42 µm		
	PET	None	Nanopillars	H	128.67 nm	Lethal to *P. aeruginosa* (~99%, 24 h)	[172]
				D	21.7 nm		
			Nanowires	H	501.4 nm		
				D	29.54 nm		

## 5. Factors Affecting Bactericidal Activity of Mechano-Bactericidal Mechanism

The stress experienced by the bacterial cell membrane due to a nanopattern is determined by both the shape and arrangement of the nanofeatures and by how strongly the cell membrane is attached to the surface, with various factors mentioned in Section 3. By fine-tuning the various geometric aspects of the nanopillars, it is possible to increase the level of stretching that the membrane undergoes [24,150,153,209]. Seng et al. showed that *S. aureus* is the most common pathogenic bacterium for breast implant infections (49% of 47 cases), whereas *P. aeruginosa* was identified as the second most pathogenic bacterium in this study (19% of 47 cases) [210]. Hence, it is more important to consider both bacteria species when developing bactericidal nanostructured surfaces. 

**Figure 11:**
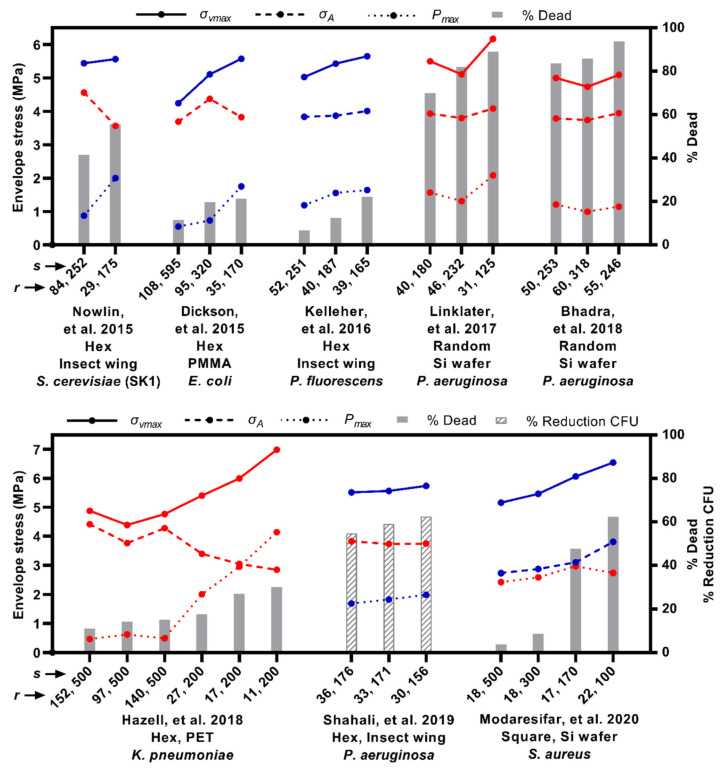
Comparison of literature results on bactericidal nanostructured surfaces demonstrating the correlation between tip radius and spacing with bactericidal efficacy. Adapted from ref. [209]. In most of the analysed studies, when the pillar experiences high von Mises stress and contact pressure, it leads to high bactericidal efficacy [21,57,101,150,154,159,161,198].

Cui et al. found that the bactericidal efficacy of nanostructures developed on PC surfaces increased with spacing and reached a maximum at 170 nm of nanopillar spacing, but then decreased. On the other hand, the bactericidal efficiency increased exponentially with the height of the nanopillar up to 300 nm, but then plateaued [195]. Different parameters of the nanostructured surface, including the shape of the feature [20,145,211], height [145,150,209], tip radius [145,209], spacing [145,209], aspect ratio [146,209], base width [209], and the arrangement (periodic or random), influence its bactericidal efficiency [207,212]. For example, Fisher et al. found that sharp diamond nanocones on a silicon wafer arranged in a nonuniform array (random) with a lower distribution density were much more bactericidal against *P. aeruginosa* than uniformly (periodic) arranged, high-density nanocones [213]. However, this cannot imply the effect of nanostructure density (or spacing) and nanoarchitecture type on bactericidal efficacy solely. Effects and relationships among the factors should be studied separately, one parameter at a time, to get a clear understanding. In addition to that, Velic et al. has summarised the effect of tip radius (or diameter) and spacing on the maximum von Mises stress $\left(\sigma_{vmax}\right)$, areal stress $\left(\sigma_{A}\right)$, and contact pressure $\left(P_{max}\right)$. These stresses were then correlated to the bactericidal efficacy from the available data in the literature (Figure 11) [209]. It was shown that the reduction in both tip radius and spacing increases bactericidal efficacy in most of the cases plotted in the graph (Figure 11). Hence, it is beneficial to use various nanostructure configurations with distinct parameters to achieve highly effective bactericidal nanostructured surfaces.

In a separate study, Velic et al. studied the effect of geometrical parameters of a nanostructured surface for lysing bacteria via a finite element analysis approach, and concluded that the reduction in nanopillar radius and spacing increased the maximal strains and the frequency of perturbation points accordingly [214]. The maximum strain is in the highest point of the pillar. This suggests that whenever there is stretching that induces pore formation and potential rupture of the bilayer, it will consistently begin within the inner leaflet at the highest point of the pillar.

Anti-bio adhesion is another mechanism which uses the wettability of the nature-inspired nanostructured surface to achieve the anti-adhesion of the bacteria [173,215]. This phenomenon is not a bactericidal activity, but it uses the non-adhesion mechanism resulting in a reduction in viability of the bacteria on the surface. There were many researchers who investigated the effect of surface wettability (hydrophobicity [154,215,216,217,218] or hydrophilicity [216,219,220]) on the bactericidal efficacy of nanostructured surfaces. Valiei et al. studied the mechano-bactericidal efficacy of etched silicon nanopillars against *P. aeruginosa* bacteria. Their results showed that the bactericidal efficacy decreases when the surfaces hydrophobicity increases. In other words, bactericidal activity was greatest on superhydrophilic surfaces and decreased as the surface became more hydrophobic. It significantly decreased when the contact angle of the substrate exceeded 90° [215]. These results suggest that super-hydrophilic nanopillared surfaces are more suitable for mechano-bactericidal activity. In contrast, superhydrophobic surfaces, although not bactericidal, may possess antibiofouling properties owing to their self-cleaning characteristics. Interestingly, according to Ivanova and colleagues, despite the cicada wing nanostructures demonstrating a superhydrophobic nature, the adhesion of bacteria was significant to kill the *P. aeruginosa* bacterium [145]. They reduced the hydrophobicity of the cicada surface by coating it with gold, which reduced the water contact angle from 158° to 105°. However, no significant difference in bactericidal action was observed, implying that the topography of the nanostructure is more important than the surface chemistry [145]. A large number of contributory factors makes it challenging to study their impact and the interactions between them. Therefore, more comprehensive studies are needed to explain the effects of those factors on the bactericidal effect of the surface.

## 6. Conclusions and Outlook

Antibacterial implants are highly desired to prevent failures due to bacterial infections. To achieve that, different approaches have been developed in the literature, briefly outlined in the Introduction. Among different pathways, “bactericidal nanostructured surfaces” have taken the spotlight in the field for years due to their excellent bactericidal efficacy on different pathogenic bacteria species, and possibility of implementation on different implant materials. Polymers are crucial materials for biomedical implants due to their inherent low density and ease of manufacturing, for example, additive manufacturing processes. TNIL, UVNIL, AAOT, EBL, and laser-based lithography processes have been efficiently used to achieve high-precision bactericidal nanofeatures on different polymeric surfaces. However, the multiple steps involved in each process make them non-feasible in the commercial scale of the relevant biomedical application. Moreover, almost all fabrication processes which were carried out in the literature were related to flat surfaces. However, most implants are not flat. Implants contain intricate shapes of internal and external features. Hence, there is no successful fabrication method to produce bactericidal nanostructures on the internal features of polymeric implants. Hydrothermal synthesis can be an effective solution to this requirement. However, the low melting point and glass transition temperature has made this process non-feasible. On the other hand, RIE and colloidal lithography are facile methods compared with lithography and templating. To optimise the topography of nanofeatures for the majority of the methods described in Table 3, assistance from the RIE process is required. However, several researchers have succeeded in fabricating nanostructures on various polymers using RIE, with or without a photoresist mask. Interestingly, these structures have shown great bactericidal efficacy against Gram-negative bacteria. However, there is no research that has shown bactericidal efficacy on Gram-positive bacteria using maskless RIE. This process has reduced most of the steps in other popular methods and could be useful in developing bactericidal nanostructures on different polymers. Due to the directional etching principle (nanofeatures will be created perpendicular to the surface) of RIE and colloidal lithography, again it is difficult to fabricate nanostructures on 3D surfaces. As a result, the density of the nanofeatures will not be homogenous due to the agglomeration of nanofeatures in low curvature areas and the surfaces which are parallel to plasma direction. In a broader context, the challenge of scaling up these processes to fabricate successful implants with strong bactericidal efficacy on a commercial scale remains unmet. Given their attributes of additive manufacturing and excellent biocompatibility, polymers are poised to play a pivotal role in addressing the aforementioned gaps in the biomedical field.

## Data Availability

No data were created in this review.
